# Mechanism of action of nadofaragene firadenovec-vncg

**DOI:** 10.3389/fonc.2024.1359725

**Published:** 2024-03-15

**Authors:** Vikram M. Narayan, Joshua J. Meeks, Jørn S. Jakobsen, Neal D. Shore, Grannum R. Sant, Badrinath R. Konety

**Affiliations:** ^1^ Department of Urology, Emory University, Atlanta, GA, United States; ^2^ Department of Urology, Northwestern University, Chicago, IL, United States; ^3^ Ferring Pharmaceuticals, International PharmaScience Center, Copenhagen, Denmark; ^4^ Carolina Urologic Research Center, Myrtle Beach, SC, United States; ^5^ Department of Urology, Tufts University School of Medicine, Boston, MA, United States; ^6^ Allina Health Cancer Institute, Minneapolis, MN, United States

**Keywords:** adenoviral-mediated interferon delivery, Adstiladrin, BCG-unresponsive, nadofaragene firadenovec-vncg, non-muscle-invasive bladder cancer, Syn3

## Abstract

Effective bladder-preserving therapeutic options are needed for patients with bacillus Calmette-Guérin unresponsive non–muscle-invasive bladder cancer. Nadofaragene firadenovec-vncg (Adstiladrin^®^) was approved by the US Food and Drug Administration as the first gene therapy in urology and the first intravesical gene therapy indicated for the treatment of adult patients with high-risk bacillus Calmette-Guérin–unresponsive non–muscle-invasive bladder cancer with carcinoma *in situ* with or without papillary tumors. The proposed mechanism of action underlying nadofaragene firadenovec efficacy is likely due to the pleiotropic nature of interferon-α and its direct and indirect antitumor activities. Direct activities include cell death and the mediation of an antiangiogenic effect, and indirect activities are those initiated through immunomodulation of the innate and adaptive immune responses. The sustained expression of interferon-α that results from this treatment modality contributes to a durable response. This review provides insight into potential mechanisms of action underlying nadofaragene firadenovec efficacy.

## 1 Introduction

Bladder cancer (BCa) is the tenth most commonly diagnosed cancer globally, with an estimated 573,278 new cases in 2020 (1). Of all BCa cases, approximately 75% are non–muscle-invasive bladder cancer (NMIBC) confined to the mucosa (stage Ta, carcinoma *in situ* [CIS]) or submucosa (stage T1) (2). High-grade (HG) tumors, accounting for approximately 31% of NMIBC cases (3), exhibit poorly differentiated cells and are more likely to progress to muscle-invasive disease (4). Guidelines by the American Urological Association, Society of Urologic Oncology, European Association of Urology, and National Comprehensive Cancer Network generally define high-risk NMIBC as CIS or HG Ta or T1 pathology (Ta ≤3 cm is considered intermediate risk) (5, 6). Individuals recently diagnosed with high-risk NMIBC have a 60–70% likelihood of recurrence and a 10–45% risk of disease progression to muscle-invasive or metastatic BCa within 5 years (6).

Although the growth of BCa is largely determined by the inherent proliferative capacity of urothelial carcinoma cells, recent advances in tumor immunology have revealed the importance of tumor-mediated immune surveillance subversion in promoting BCa growth (7, 8). Specifically, BCa has been shown to upregulate and secrete anti-apoptotic factors and immunosuppressive cytokines, such as transforming growth factor-ß, interleukin-6 (IL-6) and IL-10, to lose expression of immunogenic tumor antigens and to downregulate cell-surface major histocompatibility complex class I (MHC-I) and co-stimulatory molecules required to initiate T cell−mediated antitumor immune responses (7, 9). The cumulative effects of these immunosuppressive phenomena, coupled with the fact that the BCa microenvironment is tolerogenic and immune privileged due to the accumulation of several types of immune cells with immunosuppressive phenotypes (e.g., myeloid-derived suppressor cells, programmed death-ligand 1 (PD-L1)−expressing tolerogenic dendritic cells (DCs), and tumor-associated macrophages), result in BCa tissue being poorly immunogenic (7). By bolstering the innate and adaptive immune responses against BCa cells through vaccination with BCa-associated antigen or by administering checkpoint inhibitors of PD-L1/programmed cell death protein 1, immunotherapy attempts to induce a potent immune response to promote BCa regression. Although this goal appears straightforward, effective immunotherapy remains elusive due to problems such as T cell tolerance to BCa (self) antigen, induction of a Th2−polarized immune response, BCa heterogeneity, and limited clinical responses to immune checkpoint inhibitors (7).

Bacillus Calmette-Guérin (BCG) is a nonspecific immunotherapy that received approval by the US Food and Drug Administration (FDA) in 1990 for the treatment of patients with NMIBC (5). In clinical trials, intravesical BCG demonstrated initial complete response (CR) rates between 55% and 65% for papillary tumors and 70% and 75% for CIS (10). However, approximately one third of patients with NMIBC will not respond to BCG, and 50% will experience recurrence or progression following an initial response to BCG (11). The FDA defines BCG-unresponsive NMIBC as (1) persistent or recurrent CIS alone or with recurrent Ta/T1 (noninvasive papillary disease/tumor invades the subepithelial connective tissue) disease within 12 months of completion of adequate BCG therapy (defined as ≥5 of 6 doses of initial induction course plus either 2 of 3 doses of maintenance or ≥2 of 6 doses of a second induction course); (2) recurrent HG Ta/T1 disease within 6 months of completion of adequate BCG therapy; or (3) T1 HG disease at the first evaluation following an induction course of BCG (12). Regardless, a persistent global BCG shortage restricts access to BCG to such an extent that American Urological Association, Society of Urologic Oncology, European Association of Urology, and National Comprehensive Cancer Network guidelines have advised that BCG should be reserved for high-risk patients only (13).

The therapeutic effect of BCG is associated with the induction of Th1 immune responses. Attachment of BCG to urothelial cells triggers the cellular release of cytokines and chemokines, including IL-1, IL-6, IL-8, and tumor necrosis factor-α, which leads to the recruitment of immune cells into the urothelium. The activation of professional antigen-presenting cells, including macrophages, neutrophils, and DCs, and changes in the cytokine environment stimulate the differentiation of naïve cluster of differentiation 4^+^ (CD4^+^) T cells into Th1 and/or Th2 cells, which play respective roles in cellular and humoral immunity. Th1 cytokines, including interferon-γ (IFNγ), are associated with BCG response, and Th2 cytokines, including IL-10, are associated with BCG failure. Blocking IL-10 or inducing IFNγ can lead to a Th1-dominated immunity that is essential for BCG-mediated BCa regression (14).

While BCG-stimulated activation of the Th1 immune response demonstrates efficacy in overcoming BCa-induced immunosuppression, the development of new treatment modalities with greater immunomodulatory activity are required to improve patient outcomes (15). BCG-unresponsive NMIBC is associated with poor prognosis and historically few treatment options (6, 16). Radical cystectomy has remained the standard of care for patients with BCG-unresponsive disease and is a preferred option for high-risk disease in multiple clinical practice guidelines but is not appropriate for all patients (6, 17). For patients with high-risk, BCG-unresponsive NMIBC who are ineligible for or who choose not to undergo radical cystectomy, the therapeutic landscape has recently expanded to include intravesical chemotherapy, pembrolizumab, and the novel intravesical gene therapy, nadofaragene firadenovec-vncg (Adstiladrin^®^) (17). These treatments have all shown benefit in patients with BCG-unresponsive disease (18–20) but have notable differences in administration schedules and adverse event profiles owing to different mechanisms of action that elicit varied physiological responses. Intravesical chemotherapy encompasses multiple treatment regimens and dosing schedules and is generally well tolerated; however, it may need to be administered as often as weekly during induction therapy (17, 21). A widely used intravesical chemotherapy currently is sequential gemcitabine and docetaxel. The use of this regimen is only supported by retrospective data at present, with prospective validation still pending (18). Pembrolizumab is given via intravenous infusion every 3 to 6 weeks, and the adverse event profile includes mechanism-related immune-mediated adverse events (19, 22). Nadofaragene firadenovec is instilled intravesically on an every-3-month treatment schedule and has a well-tolerated safety profile, with micturition urgency being the most common grade 3/4 study-drug–related adverse event (20, 23). Other emerging intravesical therapies include immune adjuvants and alternative gene therapies for which the data are either emerging or pending larger phase 3 studies (16).

## 2 Gene therapy for bladder cancer

Cancer gene therapy is defined as the introduction of a therapeutic gene into a tumor cell utilizing a viral or nonviral vector. Viral vectors are widely used gene delivery vehicles in cancer therapies; adenoviruses are the preferred choice because they can express therapeutic genes episomally and have no risk of integrating into the genome (24). The deletion of *E1* and *E3* genes from the human serotype 5 adenovirus, a nonenveloped, icosahedral capsid, double-stranded DNA virus, prevents viral replication and creates space for transgenes, respectively (25). Human epithelial cells, including urothelial carcinoma cells, are particularly receptive to adenoviral infection due to the ubiquitous expression of the coxsackie/adenovirus receptor (26). Adenoviruses interact with the coxsackie/adenovirus receptor, leading to intracellular incorporation of the virus and subsequent expression of the transgene; once translated, the resultant protein remains detectable for up to 10 days after adenoviral infection (27, 28).

The urothelium of the bladder is a complex, multilayer surface that acts as a barrier to pathogens and urinary waste products. Efficient viral transduction of the urothelium requires a robust means to permeate the protective glycosoaminoglycan layer of the bladder mucosa. Investigation into the structure of Big CHAP (N,N’-Bis(3-D-gluconamidoproply)cholamide), a nonionic detergent used as a transduction-promoting agent in early intravesical adenoviral vector studies, led to the discovery of Syn3, a polyamide surfactant and synthetic excipient that promotes adenoviral transduction across the glycosoaminoglycan layer of the inner wall of the bladder (10, 29, 30). The simultaneous administration of adenovirus in a formulation containing Syn3 markedly increased adenoviral-mediated gene transfer and expression, not only in the normal urothelial cells but also in human superficial transitional cell carcinoma growing within the bladder of athymic nude mice (30). Syn3 is an important additive for adenoviral-mediated gene transfer for the treatment of NMIBC by intravesical administration (31).

**Figure 1:**
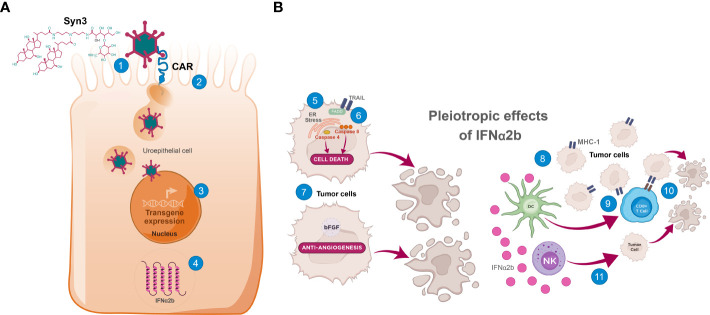
The mechanism of action of nadofaragene firadenovec. **(A)** Internalization of adenoviral vector into uroepithelial cells and transcription/translation of IFNα2b. (1) Syn3 promotes adenoviral vector access to uroepithelial cells (29); (2) After CAR-mediated endocytosis into the uroepithelial cell and escape from endosomes, the adenoviral capsid translocates to the nuclear envelope where it disassembles, and the human IFNα2b transgene is imported into the nucleus (26); (3) After import into the nucleus, IFNα2b cDNA is transcribed into mRNA; (4) IFNα2b mRNA is translated, leading to sustained production of IFNα2b (27, 28, 30). **(B)** Summary of putative direct and indirect effects of IFNα2b. IFNα2b exerts pleiotropic antitumor effects directly and indirectly in the tumor microenvironment (32). Direct cytotoxic effects include (5) induced ER stress in bladder cancer cells, leading to caspase 4 activation and cell death (33), (6) increased expression of TRAIL, leading to caspase 8 activation and cell death (34), and (7) antiangiogenic effects through downregulation of growth factors including bFGF (35, 36), leading to tumor hypoxia and central necrosis. Direct immunomodulatory effects are elicited through (8) upregulation of the presentation of surface tumor-associated antigens via augmentation of MHC-I class molecules, increasing the immunogenicity of tumor cells (37). Indirect immunomodulatory effects occur through (9) stimulation of DC-priming of cytotoxic CD8^+^ T cells, which (10) kill MHC-I^+^ tumors, and (11) increased antitumor activity of NK cells, which preferentially kill BCa cells lacking MHC-I (downregulation or loss of MHC-I is common in BCa cells) (32). BCa, bladder cancer; bFGF, basic fibroblast growth factor; CAR, coxsackievirus and adenovirus receptor; CD, cluster of differentiation; cDNA, complementary deoxyribonucleic acid; DC, dendritic cell; ER, endoplasmic reticulum; FADD, Fas-associated protein with death domain; IFNα2b, interferon α2b; MHC-I, major histocompatibility complex class I; mRNA, messenger ribonucleic acid; NK, natural killer; TRAIL, tumor necrosis factor−related apoptosis-inducing ligand.

Nadofaragene firadenovec-vncg received FDA approval as the first gene therapy in urology and the first intravesical gene therapy indicated for the treatment of adult patients with high-risk BCG-unresponsive NMIBC with CIS with or without papillary tumors (23). Nadofaragene firadenovec is a nonreplicating adenoviral vector−based gene therapy that delivers human *IFNα2b* complementary DNA to urothelial cells and Syn3 to enhance viral transduction of the urothelium. *IFNα2b* complementary DNA is transcribed into IFNα2b protein in bladder epithelial cells, where it exhibits direct and indirect immunomodulatory effects inhibiting tumor growth (28) (**Figure 1**).

IFNα has been used as a monotherapy and in combination with other agents, including BCG (38–40). Phase 2 studies of intravesical IFNα2b monotherapy at doses of 50–100 MIU have shown CRs, albeit of short duration, in up to 40% of patients with NMIBC, with most patients relapsing within 1 year (41). Limitations observed with intravesical IFNα2b therapy are likely due to a short drug exposure time rather than a lack of inherent antitumor activity (42). Local gene delivery with nadofaragene firadenovec can maximize transgene expression in the urothelium with prolonged local exposure of tissues to IFNα while minimizing systemic adenovirus distribution. In an orthotopic model of human BCa in nude mice to test the delivery and efficacy of nadofaragene firadenovec, high urinary IFN levels and marked tumor regression were observed following treatment (30). Adenoviral interferon-alpha 2b (AdIFNα) treatment also had cytotoxic effects on cells that were previously shown to be resistant to recombinant human IFNα (rhIFNα), which was attributed to a strong bystander effect in neighboring cells that potentially targeted tumor cells that were not effectively transduced during their initial exposure. Elevated bladder tissue IFNα levels were observed for ≥7 days following nadofaragene firadenovec therapy compared with rhIFNα levels and declined rapidly after treatment (30). This important discovery emphasized the benefit of gene therapy in overcoming the issue of durability with intravesically administered rhIFNα.

In a multicenter phase 2 trial, 40 patients with HG BCG-refractory or relapsed NMIBC were randomly assigned to receive either low- or high-dose intravesical nadofaragene firadenovec; patients who responded at 3, 6, and 9 months were re-treated at 4, 7, and 10 months (43). The primary endpoint was freedom from HG disease recurrence at 12 months (defined by a negative result on for-cause or end-of-study biopsy), which was found to be similar between the two dose groups, with 7 patients (33%) and 7 patients (37%) achieving 12-month recurrence-free survival of HG disease in the low- and high-dose groups, respectively. In subgroup analyses, 50% of patients with papillary disease and 30% of patients with CIS had 12-month recurrence-free survival. The most common adverse (AEs) events were lower urinary tract symptoms; no grade 4 or 5 AEs occurred and no patient discontinued therapy due to an AE. The higher dose was selected for use in the subsequent multicenter phase 3 trial (20). A total of 53.4% of patients with CIS with or without HG Ta/T1 had a CR at the 3-month assessment visit; this response was maintained through 12 months in 45.5% of these patients. In the HG Ta/T1 cohort, 72.9% of patients had a CR at 3 months, with 43.8% remaining recurrence-free through 12 months. The most frequently reported drug-related AEs were discharge around the catheter during instillation, fatigue, bladder spasms, and micturition urgency. Most AEs were transient and classified as either grade 1 or 2. There was a low discontinuation rate (1.9%), and no patient died from treatment-related AEs during the 12-month follow-up period (20). A major strength of the study was its mandatory end-of-study biopsy at 12 months, which provided objective pathologic disease assessment as opposed to visual assessments alone. Additionally, in the CIS cohort, patients who had a CR had a significantly longer median time to cystectomy of 11.4 months, compared with only 6.4 months among those who did not.

Because the human population is largely seropositive for anti-adenovirus antibodies, a planned secondary analysis of the phase 3 data investigated whether anti-adenovirus antibody levels predicted the durability of response to nadofaragene firadenovec (44). Baseline titer levels did not predict treatment response, suggesting that pre-exposure to circulating adenovirus did not adversely affect the efficacy of nadofaragene firadenovec. A 3-month adenovirus titer level of >800 was associated with a higher likelihood of durable response, and peak posttreatment titer levels of >800 were noted in 89% of responders versus 59% of non-responders. Although reasons for an increase in anti-adenovirus antibody titer levels observed in durable responders remain to be fully elucidated (44, 45), serum anti-adenovirus antibodies may serve as a predictive marker for nadofaragene firadenovec response durability.

For patients with BCG-unresponsive NMIBC who prefer a bladder-preserving treatment approach, nadofaragene firadenovec offers a compelling novel treatment option. After its FDA approval in December 2022, nadofaragene firadeneovec was initially available to a limited number of clinical study sites and community clinics due to supply constraints. The manufacturing processes were subsequently optimized and as of January 2024, the product has become fully available across the United States for healthcare providers to prescribe for appropriate patients (46). As of February 2024, completion of construction is near final for two new, state-of-the-art facilities that will be dedicated to manufacturing of nadofaragene firadenovec for future long-term supply.

## 3 Mechanism of action of nadofaragene firadenovec

The antitumor efficacy of AdIFNα results from the direct and indirect pleiotropic antitumor effects of IFNα. All cell types, including DCs, produce IFNα in response to the presence of cancer cells or other immune stimuli. Interferons modulate direct and indirect antitumor activities, typically through their canonical signaling via the Janus kinase (JAK)/signal transducer and activator of transcription (STAT) pathway. IFNα, part of the IFN-I family, signals through the IFNα receptor (IFNAR). IFN-I binds a heterodimeric receptor formed by IFNAR1 and IFNAR2 chains, causing their respective, constitutively associated JAKs, TYK2 and JAK1, to activate and phosphorylate STAT1 and STAT2. Phosphorylated (p)-STAT1 and p-STAT2 bind to IFN regulatory factor 9 and form a transcriptional complex named IFN‐stimulated gene factor 3, which is recruited to the IFN-stimulated response elements and regulates the transcription of downstream IFN‐stimulated genes. Key IFN‐stimulated genes include IFNα-inducible protein 27, PD-L1, and tumor necrosis factor–related apoptosis-inducing ligand (TRAIL) (32, 47, 48).

IFNα exhibits direct cytotoxic effects through endoplasmic reticulum stress, caspase 4 activation (33), and induction of TRAIL expression (34, 49). In BCa cells, rhIFNα-induced TRAIL expression resulted in cell death via an IFN regulatory factor-1−dependent mechanism (34), which may be a key cell death pathway underlying direct IFNα activity, because elevated TRAIL levels have been found in patients with detectable urinary IFNα following transduction (50). IFNα also mediates an antiangiogenic effect (35, 36, 51); preclinical studies have shown that systemic administration of rhIFNα to bladder tumor−bearing mice was associated with decreased angiogenic factors, including basic fibroblast growth factor (35). In addition, IFNα can directly upregulate the presentation of surface tumor−associated antigens via augmentation of MHC-I molecules, increasing the immunogenicity of tumor cells and making them more vulnerable to identification and subsequent destruction by cytotoxic CD8^+^ T cells (37). Increased tumorigenicity is especially important in BCa cells, which are renowned for immune evasion (7).

The indirect effects of IFNα, which include tumor microenvironment immunomodulation through the enhanced proliferation, maturation, and antigen presentation of immune cells such as DCs, macrophages, and natural killer (NK) cells, strengthen innate and adaptive antitumor immunity. IFNα is involved in antigen recognition and processing, leading to CD8^+^ T cell, NK cell, and DC activation. IFNα further promotes the adaptive antitumor response by stimulating the DC-priming of CD8^+^ T cells. In addition, stimulation of the adaptive immune response by type I IFNs is expected to complement immune checkpoint blockade. Together, the direct and indirect IFN-α2b effects can lead to BCa cell lysis and the release of BCa-associated antigen, further enhancing BCa immunogenicity and subsequent tumor regression (32, 48).

IFNα also augments the antitumor activity of NK cells, which preferentially kill MHC-I−deficient cells. Activated NK cells recognize and directly attack cancer cells by releasing cytotoxic granules containing perforin and granzymes. These substances create pores in the cancer cell membrane and induce apoptosis. Because downregulation or total loss of MHC-I expression is common in BCa cells (52), NK cells likely play a significant role in the antitumor immune activity of IFNα in BCa (32).

Taken together, these studies postulate that MHC-I−deficient BCa cells transduced with nadofaragene firadenovec produce IFNα2b, which may modulate NK cell responses by upregulating stress-induced ligands for activating NK cell receptors and promoting NK cell priming by DCs. Concurrently, MHC-I−expressing BCa cells transduced by nadofaragene firadenovec produce IFNα2b which may augment anti-BCa CD8^+^ T cell responses by upregulating MHC-I, promoting antigen presentation, and activating signaling pathways that augment T cell proliferation and cytotoxicity (32).

## 4 Conclusions

Nadofaragene firadenovec is the first FDA-approved gene therapy for high-risk BCG-unresponsive NMIBC with CIS with or without papillary tumors. The mechanism of action of nadofaragene firadenovec is hypothesized to result via direct and indirect IFNα2b activity. IFNα induces cell type-specific direct biological responses, including apoptosis and angiogenesis inhibition, affecting tumor cell initiation and progression. Indirect activity occurs through immunoregulation, by stimulating immune cells, including NK and T cells, and increasing antigen presentation by macrophages and DCs, which augments a more robust immune response.

## Author contributions

VN: Conceptualization, Funding acquisition, Project administration, Supervision, Writing – original draft, Writing – review & editing. JM: Conceptualization, Writing – original draft, Writing – review & editing. JJ: Conceptualization, Writing – original draft, Writing – review & editing. NS: Conceptualization, Writing – original draft, Writing – review & editing. GS: Conceptualization, Writing – original draft, Writing – review & editing. BK: Conceptualization, Funding acquisition, Project administration, Supervision, Writing – original draft, Writing – review & editing.
